# Leptin pharmacokinetics in male mice

**DOI:** 10.1530/EC-16-0089

**Published:** 2016-12-20

**Authors:** Robert A Hart, Robin C Dobos, Linda L Agnew, Neil A Smart, James R McFarlane

**Affiliations:** 1 Centre for Bioactive Discovery in Health and AgeingUniversity of New England, Armidale, New South Wales, Australia; 2 NSW Department of Primary IndustriesArmidale, New South Wales, Australia

**Keywords:** leptin, pharmacokinetics, gastrointestinal tract, dimorphism, mice

## Abstract

Pharmacokinetics of leptin in mammals has not been studied in detail and only one study has examined more than one time point in non-mutant mice and this was in a female mice. This is the first study to describe leptin distribution over a detailed time course in normal male mice. A physiologic dose (12 ng) of radiolabelled leptin was injected into adult male mice via the lateral tail vein and tissues were dissected out and measured for radioactivity over a time course of up to two hours. Major targets were the digestive tract, kidneys, skin and lungs. The brain was not a major target, and 0.15% of the total dose was recovered from the brain 5 min after administration. Major differences appear to exist in the distribution of leptin between the male and female mice, indicating a high degree of sexual dimorphism. Although the half-lives were similar between male and female mice, almost twice the proportion of leptin was recovered from the digestive tract of male mice in comparison to that reported previously for females. This would seem to indicate a major difference in leptin distribution and possibly function between males and females.

## Introduction

Leptin is a cytokine hormone released into circulation from adipose tissue, placenta and stomach, and is best known for its effects on energy balance (1, 2, 3, 4, 5). Animals that lack leptin (*ob/ob*), or its long receptor, LepRb (*db/db*), display a phenotype with voracious appetite and obesity (1, 6, 7, 8), while correcting the deficiency ameliorates the abnormal phenotype (9, 10). Leptin signals via its receptors, notably LepRb, which is capable of activating the JAK-STAT pathway (11, 12) and is found at high density in the hypothalamus (8). Leptin is known to suppress appetite and is thought to elicit this change in behaviour by modulating the expression of other appetite-modulating hormones such as neuropeptide Y and proopiomelanocortin (13, 14). As leptin circulates at concentrations correlating with adiposity (15), it has been proposed that it enters the brain from the periphery to signal information about energy balance to the central nervous system. This signalling allows a relatively stable body weight to be maintained and in most cases of obesity a surfeit of leptin is found due to leptin resistance (5).

Leptin has a number of diverse roles in the periphery, many of them related to energy balance. In skeletal muscle, leptin is known to stimulate energy expenditure and increase the oxidation of fatty acids, while reducing the uptake of glucose (16); whereas in various tissues including bone (17), the gut (18) and lungs (19) leptin has been shown to regulate maturation and development. Leptin is also involved in the regulation of immune function (20) and may act to regulate appetite from the lumen of the digestive tract via the afferent vagus nerve (21, 22). Leptin is critical in reproduction, allowing pubertal development (23, 24) as well as regulating testis development (25) and ovarian follicle development (26).

Leptin is known to circulate at higher concentrations in females than males in comparable body condition, suggesting that there may be some sexual dimorphism in the roles it plays and possibly the tissues it targets. In women, leptin circulates at much higher concentrations with reports ranging from 40 to 300% higher than that found in men (27, 28), similar trends have been observed in laboratory monkeys (29) and mice (30). In females, leptin is important for reproduction and is necessary for fertilisation, implantation in the endometrium and development of the conceptus (31). In males, the roles for leptin are less clear, as at high concentrations it correlates with an attenuation of testosterone secretion (32), while in younger males leptin facilitates development and maturation (25). To date, distribution of peripheral leptin has not been compared in males and females, a comparison which may allow the examination of possible sexual dimorphism of leptin functions.

Despite the large volume of research on leptin, its total distribution is not well characterised. Previous studies have only examined the distribution of leptin at one or two time points, or over time in a single tissue, with the exception of previous work by the authors, which examined in female mice a number of tissues over a time course (33).

## Materials and methods

### Animals

Random-bred male Swiss albino mice, approximately 12 weeks of age, were obtained from a colony maintained in the University of New England Small Animal House and kept in same-sex litter mate groups of up to 6 individuals. Mice had *ad libitum* access to commercial chow and tap water. A 12 h light–darkness cycle was maintained with lights on at 0700 AEST and temperature was maintained at 22 ± 0.5°C. All work was approved by the University of New England Animal Ethics Committee and conformed to the NHMRC Code of Practice for the Care and Use of Animals for Scientific Purposes.

### Experimental protocol

Recombinant human leptin (R&D Systems) was labelled with ^125^Iodine (Perkin Elmer) using the Iodogen method (Thermo Scientific). Mice were injected intravenously via the lateral tail vein with 12 ng (14.4 kBq) leptin in a total volume of 100 µL made up with phosphate-buffered solution. Animals were then placed in an individual cage with access to food and water until the specified time when the animal was killed by rapid CO_2_ asphyxiation with *n* = 4 for each time point, to observe distribution over a time course. Tissues were dissected and weighed with duplicate samples placed in polypropylene tubes and measured for total γ-radioactivity (1470 Wizard, Perkin Elmer). Background radiation was subtracted from all samples. Due to equipment failure, no data were collected for brain or digestive tract at 120 min.

Blood was collected via cardiac puncture and transferred to vial containing 20 IU heparin and measured in duplicate. Total blood volume was calculated as 84.7 mL/kg body mass as previously reported (34). The skin was removed apart from that around the snout and ‘cuffs’ around the limbs and weighed intact. Four samples were taken from the left forelimb, right hindlimb, interscapular and dorsal cervical regions. Gut tissues were measured with contents.

### Data analysis

Radioactivity was measured in duplicate samples and the mean values were multiplied across the weight of the respective intact tissue to calculate total radioactivity. All data presented are mean ± s.e.m. unless otherwise indicated.

Plasma clearance of leptin was calculated by using the area under the curve method, fitting a second-order exponential decay curve to the respective data using Origin 7.0300 (35) using the formula: where *y* represents the radioactivity per mL of blood or total radioactivity recovered from the body at time *x* (min), *A* and *B* are the radioactivity present in each pool, and *t*_1_ is 1/*α* and *t*_2_ is 1/*β* where *α* and *β* are the decay constants for the respective pools.

## Results

After intravenous administration of radiolabelled leptin to male mice tissues were examined for radioactivity. The major targets as defined by total radioactivity were the liver, kidneys, digestive tract (with contents), blood and skin. Other tissues examined were pooled as ‘other tissues’ and included the brain, submandibular salivary glands, spleen, heart, lungs, testes, epididymides, epididymal fat and seminal vesicles.

**Figure 1:**
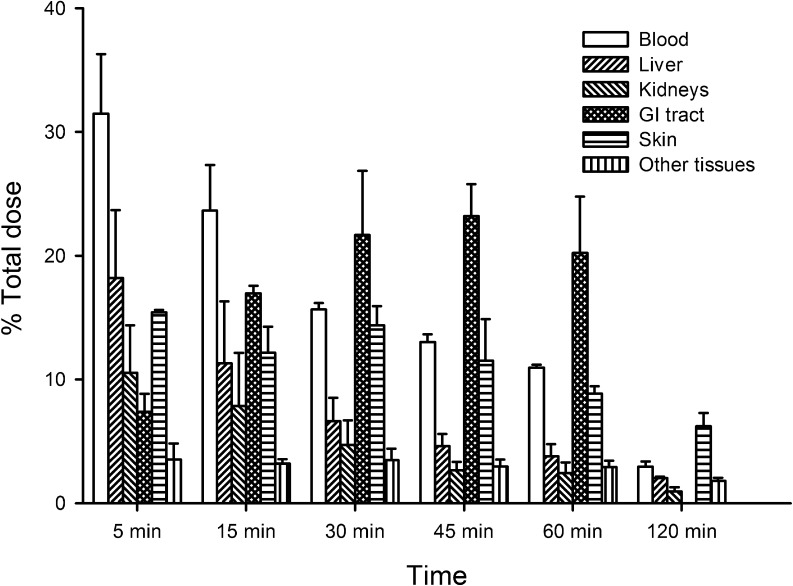
Major targets for administered radiolabelled leptin in male mice measured as percentage of dose (GI Tract – gastrointestinal tract and contents; Other tissues – pooled data for testes, epididymides, seminal vesicles, epididymal fat, heart, lungs and brain).

Over the course of the experiment, the total recovery of administered leptin decreased from 78.83 ± 17.84% of the dose 5 min after administration to 13.90 ± 2.10% 120 min after administration. Of the major targets, the liver, kidneys and skin, as well as the blood, showed rapid clearance in the total amount of radiolabelled leptin recovered, and 5 min after administration each of these contained >10% of the administered dose and collectively accounted for approximately 70% of the administered dose (Fig. 1). Radiolabelled leptin recovered from the blood declined over the duration of the experiment to 2.94 ± 0.42% 120 min post-injection. The amount of administered leptin recovered from the pooled ‘other tissues’ appeared relatively stable at approximately 3% of the dose over the first hour examined followed by a decrease to 1.81 ± 0.24% of the dose 120 min post-injection. Within the ‘other tissues’ the epididymal fat showed an increase in radiolabelled leptin recovered 15–30 min after administration from 0.54 ± 0.09% to 1.05 ± 0.05% of the total dose. Recovery then declined to 0.43 ± 0.15% of the total dose 45 min post-administration, there was then a gradual increase to 0.62 ± 0.06% of the total dose 120 min post-injection (data not shown). The most radiolabelled leptin recovered from the brain was 0.15 ± 0.05% of the total dose, 5 min after administration. Radiolabelled leptin recovered from the digestive tract tissues and contents increased from 7.38 ± 1.45% of the dose 5 min post-injection to 23.20 ± 5.16% 45 min post-injection, before a slight decline to 20.22 ± 4.55% of the dose 60 min after administration.

**Figure 2:**
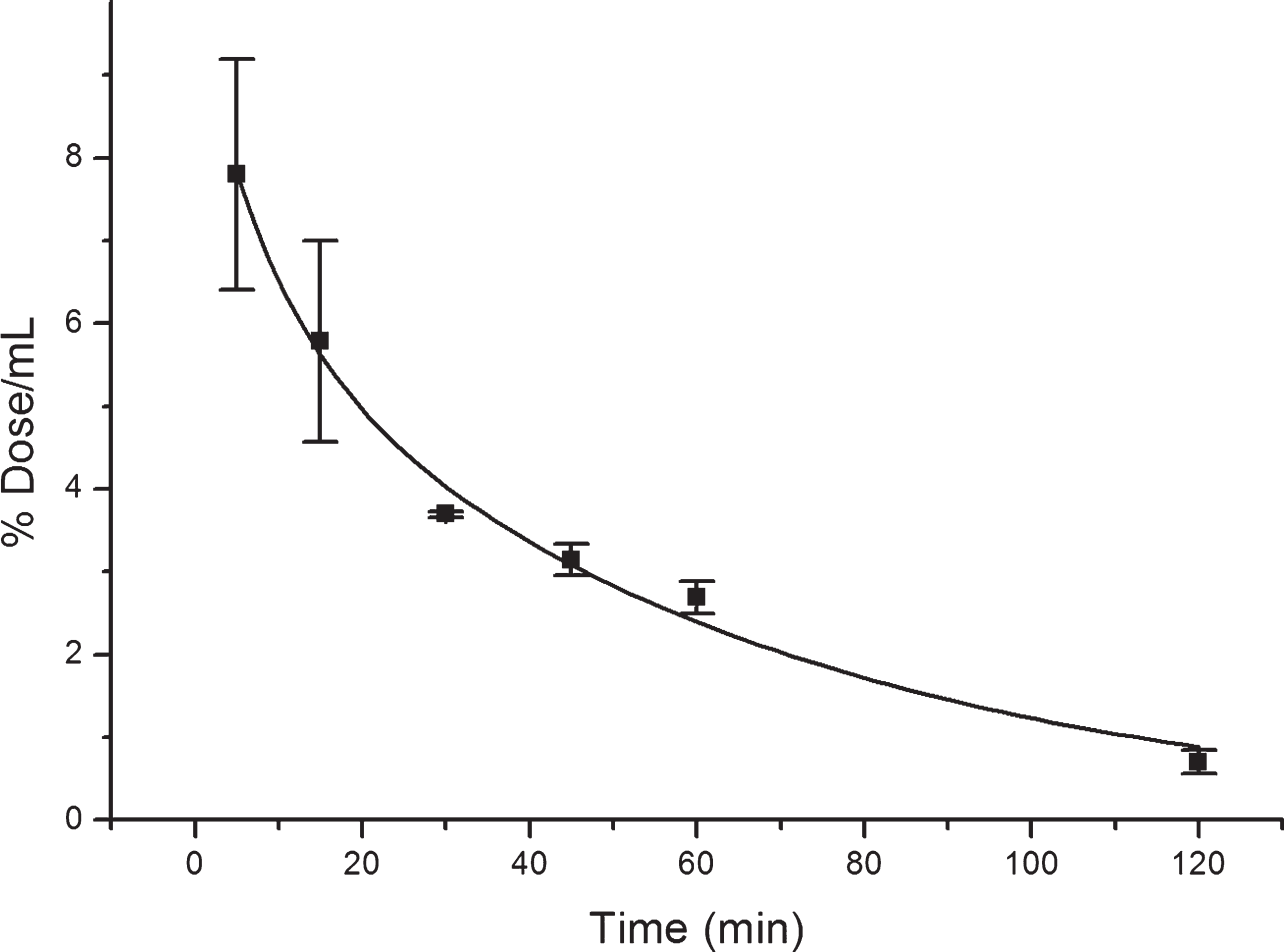
Plasma clearance of intravenously administered radiolabelled leptin in male mice with a second-order exponential decay curve fit.

The amount of radiolabelled leptin recovered per mL (% dose/mL) of blood rapidly decreased from 7.80 ± 1.40% dose/mL 5 min post-injection to 5.78 ± 1.21% dose/mL 15 min after injection. Apart from slight decrease between 30 and 45 min after administration from 3.69 ± 0.03% dose/mL to 3.14 ± 0.19% dose/mL, a steady decrease was then observed to 0.70 ± 0.14% dose/g 120 min post-injection (Fig. 2). After fitting a second-order exponential decay curve to these data, plasma clearance rate of administered leptin was calculated to be 5.46 mL/kg/min and plasma half-life to be 29.7 min with an α phase half-life of 6.14 min and a β phase half-life of 41.4 min.

**Figure 3:**
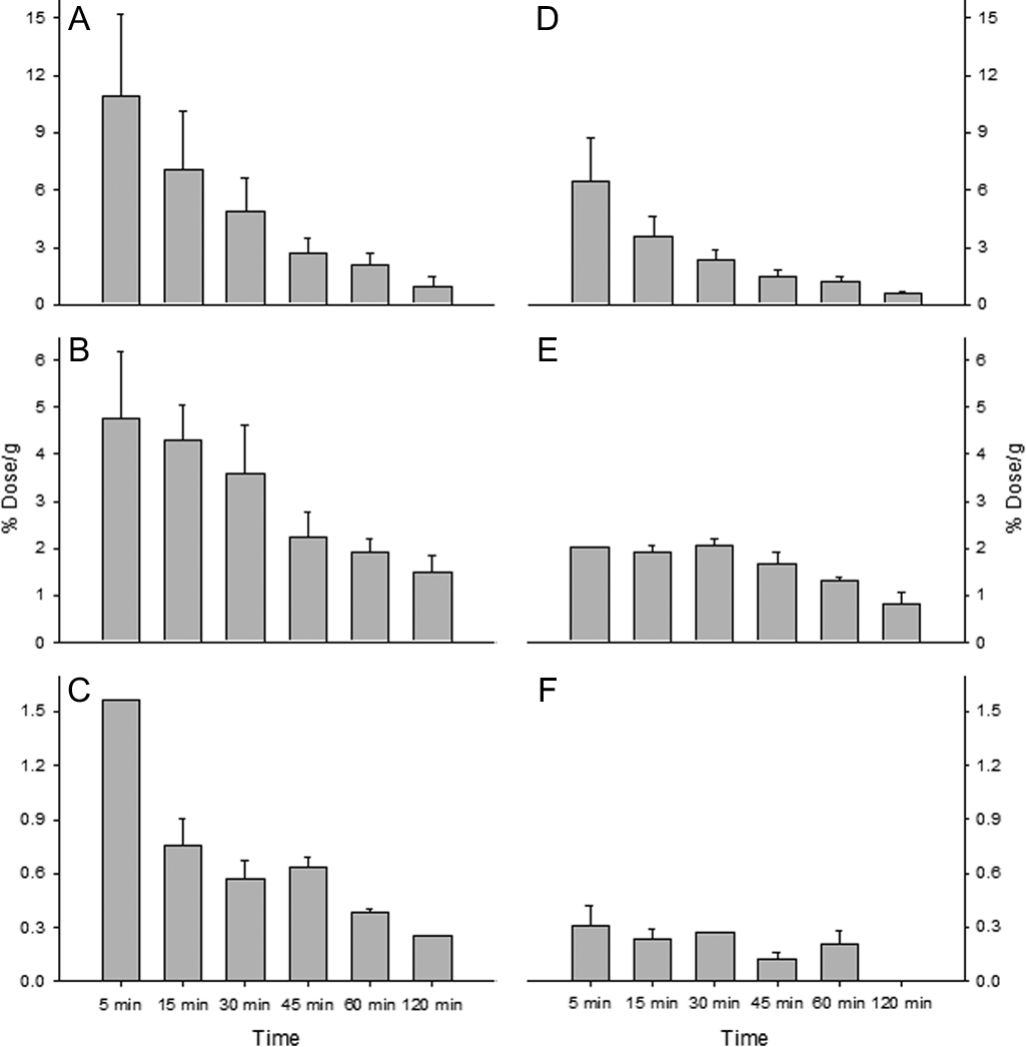
Radiolabelled leptin recovered per gram of tissue from male mice (A – kidneys; B – lungs; C – muscle; D – liver; E – skin; F – brain).

Radiolabelled leptin recovered per gram (or mL) of tissue revealed major sites of accumulation, which were the blood (Fig. 2), liver, kidneys and lungs (Fig. 3). Each of these tissues displayed a decrease in radiolabelled leptin per gram over the duration of the experiment with the highest measured concentrations 5 min post-injection with the kidneys having 10.91 ± 4.33% dose/g, liver 6.42 ± 2.31% dose/g and the lungs 4.75 ± 1.43% dose/g. The recovery per gram of skin remained at approximately 1.3–2.05% dose/g up to 60 min post-injection, decreasing to 0.82 ± 0.23% dose/g 120 min after administration. Radiolabelled leptin recovered from skeletal muscle of the thigh (quadriceps and biceps femoris) declined from 0.75 ± 0.14% dose/g 15 min after injection to 0.38 ± 0.17% dose/g 60 min after administration. The highest recovery from the brain was 0.31 ± 0.11% dose/g, 5 min after administration.

## Discussion

This report contains the most comprehensive time course study of leptin in male mice after intravenous administration of a physiologic dose performed to date. The major targets in terms of total radiolabelled leptin were the liver, kidneys, digestive tract (with contents) and skin. In terms of radiolabelled leptin per gram of tissue the lungs were also considered a major target. The brain was not a major target for administered leptin by either measure at any time examined. The distribution reported here for males differs from that reported previously in females, indicating sexual dimorphism.

Similar proportions of radiolabelled leptin were recovered from the skin of male mice to that previously recorded in female mice. It should be noted that in both studies the way the skin was removed means that it may also be a proxy for subcutaneous fat. Here, 15.42% of the dose was recovered from the skin of male mice 5 min after administration, while in females this was 11.43% (33), and the tissue distribution, in terms of dose per gram, is similar to that reported in female rats (36). In the lungs of male mice, a relatively low total amount of 1.18% of the dose was recovered 5 min post-injection (data not shown), but in terms of radiolabelled leptin per gram the lungs had the second highest measurements 15–60 min following administration, which may be slightly higher than reported in female rats (36). Interestingly, the dose per gram pattern seen in the lungs of males differed from that of females in that the females initially showed a higher recovery followed by a rapid clearance (33), whereas the males displayed a slower clearance.

The amount of radiolabelled leptin recovered from the brain of male mice 5 min post-injection (0.15% dose) was proportionally less than that was found in female mice (0.27% dose) 5 min after administration (33). Similarly, the amount of radiolabelled leptin per gram of brain tissue was higher in females (0.55% dose/g vs 0.31% dose/g in males). This may have been due to the same fixed dose (12 ng) being given to both males and the smaller females. However, the rate of uptake into the brain may differ between the sexes. Accordingly, if leptin re-enters the circulation after entering the brain, these data may partially explain the observation in humans, where women had a higher net overflow of leptin from the brain than similar sized men (37). More investigation would be needed to explore this possibility in detail.

The total radiolabelled leptin recovered in this study (up to 78.83%) was much less than that was found for female mice (up to 95.33%) (33). The reason for this discrepancy is not clear, but may be an indicator of sexual dimorphism in leptin distribution. Part of the unrecovered dose is thought to be within the musculoskeletal system, as radiolabelled leptin was recovered from the muscle. The musculoskeletal system was not extensively sampled as it was not expected to be a major target, but male mice are assumed to have 30% of their body mass as muscle (38). Assuming a recovery of approximately 0.60% dose/g in muscle, an intermediate value to those reported 15–60 min post-injection from thigh muscle, then the mice in this study may have had approximately 8.51% of the dose sequestered in muscle. This recovery would be almost twice what was estimated for females at 4.55% using similar calculations (33). The reason for this is not clear; however, as leptin stimulates the oxidation of fuel in skeletal muscle (39), the higher concentration of circulating leptin entering skeletal muscle may contribute to the lower proportion of body fat observed in males. Furthermore, as leptin is known to exert a haematopoietic effect (40), bones may also represent a potential sink for unrecovered leptin, which may warrant further investigation.

The total plasma half-life for radiolabelled leptin was found to be 29.7 min in male mice. This is slightly less than what we reported in female mice (32.0 min) (33) and similar to the reported endogenous leptin half-life of 24.9 min in humans (41). The plasma α and β half-lives reported, 6.1 and 41.4 min respectively, are similar to previous findings in rats of α half-lives of 3.4–5.1 min and β half-lives of 49–90 min (36, 42, 43). In contrast, we found that in female mice the β half-life was 230.1 min (33) and both reported values are lower than has been reported for human leptin after administration to rhesus monkeys, where the α and β half-lives were 10.4 and 96.4 min, respectively. However, it is worth noting that in two of these studies doses >0.25 mg/kg were administered (43, 44), which may have altered the normal clearance of leptin, as has been shown for other hormones (45).

The proportion of the dose of radiolabelled leptin in the blood in male mice was 31.47%. This was lower than those found in female mice, where 42.91% of the dose was found in the blood, which may be due to the larger size of the male mice (45.6 ± 0.9 g vs 37.9 ± 2.3 g). The higher plasma clearance rate found in male mice (5.46 mL/min/kg) than female mice (1.59 mL/min/kg) may also have contributed to these differences. The proportion of the dose recovered from the kidneys was similar in male and female mice, at approximately 11% 5 min post-injection; however, this equates to 10.91% of the dose/g in males, vs 22.71% of the dose/g in females (33). In humans it has been shown that males have a higher clearance rate of leptin (141 mL/min vs 91 mL/min), which has been hypothesised to be due to increased renal plasma flow (46). However, in mice it may also be that there is greater access to LepR in the kidneys of male mice, as hypothesised for rats (47).

The liver, kidneys and lungs exhibited similar clearance profiles to that of the blood. These tissues are well perfused and may contain approximately 20% plasma by mass (48). Therefore, we concluded that at least 80% of the radiolabelled leptin recovered in these tissues was tissue specific rather than blood borne.

The reported lower total recovery of radiolabelled leptin from the blood of male mice in comparison with female mice is consistent with reports of sexual dimorphism of leptin plasma concentrations. This pattern of plasma concentration has been reported in humans (27, 28, 49, 50), mice (30) and monkeys (29), although several studies have reported that male rodents exhibit higher plasma concentrations than females (51, 52). The whole body half-life of radiolabelled leptin for males was found to be longer than for females at 57 min vs 47.3 min (33). As discussed, the muscle may accumulate a significant portion of radiolabelled leptin, this may also be true of the adipose tissue and the bones, as indicated by low levels of accumulation in distribution studies in several species (36, 53, 54). The epididymal fat (contained within the ‘other tissues’) showed a decline of 0.61% of the administered dose 30–45 min post-injection, coinciding with the very minor drop in detected radiolabelled leptin in the blood. This may be indicative of tissue uptake and subsequent release of leptin, but further investigation would be needed to confirm this.

The digestive tract was identified as a major target for radiolabelled leptin in male mice. Previously, in females up to 12.80% of the administered dose was found in the digestive tract (33), whereas here in males up to 23.20% of the dose was recovered from the digestive tract. In both females and males, the amount of radiolabelled leptin recovered increased over the first hour of the experiment, before declining slightly 120 min after administration. This seems to indicate that leptin has a major role in the gastrointestinal tract and that this role may be more prominent in males. As the radiolabelled leptin accumulated in the digestive tract over time, it seems possible that circulating leptin may enter the digestive tract to elicit a response. Leptin is known to be a secretion of the stomach (3) and remains stable in gastric juices, while leptin receptors are expressed in the stomach (55) and small intestine at both the luminal and basolateral surfaces (56). In the small intestine, leptin modulates the absorption of sugars (57, 58) and peptides (59), while in the hindgut leptin may inhibit infection (60, 61). Although some potential roles for leptin have been identified in the digestive tract, the fate of any leptin in the lumen has not been examined to date and may provide further insight into potential roles for leptin in the gastrointestinal tract.

Radiolabelled leptin distribution was examined in male mice following a physiologic intravenous dose. Major targets included the liver, kidneys, skin and digestive tract, and a high concentration was found in the lungs. This is similar to findings in female mice, but some major differences seem to indicate that profound dimorphism of distribution exists between the sexes. Notably, almost twice the proportion of administered leptin was recovered from the digestive tract of male mice versus female mice. The half-life of leptin in male mice was shorter than that reported for female mice and male mice also had a higher leptin plasma clearance and lower leptin recovery from blood versus females.

## Declaration of interest

The authors declare that there is no conflict of interest that could be perceived as prejudicing the impartiality of the research reported.

## Funding

This research did not receive any specific grant from any funding agency in the public, commercial or not-for-profit sector.
